# Extent of Ischemic Brain Injury After Thrombotic Stroke Is Independent of the NLRP3 (NACHT, LRR and PYD Domains-Containing Protein 3) Inflammasome

**DOI:** 10.1161/STROKEAHA.118.023620

**Published:** 2019-04-08

**Authors:** Eloise Lemarchand, Jack Barrington, Alistair Chenery, Michael Haley, Graham Coutts, Judith E. Allen, Stuart M. Allan, David Brough

**Affiliations:** 1 From the Division of Neuroscience and Experimental Psychology (E.L., J.B., G.C., S.M.A., D.B.), School of Biological Sciences, Faculty of Biology, Medicine and Health, Manchester Academic Health Science Centre, University of Manchester, United Kingdom.; 2 Division of Infection, Immunity and Respiratory Medicine (A.C., M.H., J.E.A.), School of Biological Sciences, Faculty of Biology, Medicine and Health, Manchester Academic Health Science Centre, University of Manchester, United Kingdom.; 3 Lydia Becker Institute of Immunology and Inflammation (E.L., J.B., A.C., M.H., G.C., J.E.A., S.M.A., D.B.), School of Biological Sciences, Faculty of Biology, Medicine and Health, Manchester Academic Health Science Centre, University of Manchester, United Kingdom.

**Keywords:** brain, cytokine, inflammasome, inflammation, interleukin

## Abstract

**Background and Purpose—:**

A major process contributing to cell death in the ischemic brain is inflammation. Inflammasomes are multimolecular protein complexes that drive inflammation through activation of proinflammatory cytokines, such as IL (interleukin)-1β. Preclinical evidence suggests that IL-1β contributes to a worsening of ischemic brain injury.

**Methods—:**

Using a mouse middle cerebral artery thrombosis model, we examined the inflammatory response after stroke and the contribution of the NLRP3 (NACHT, LRR and PYD domains-containing protein 3) inflammasome to ischemic injury.

**Results—:**

There was a marked inflammatory response after stroke characterized by increased expression of proinflammatory cytokines and NLRP3 and by recruitment of leukocytes to the injured tissue. Targeting NLRP3 with the inhibitor MCC950, or using mice in which NLRP3 was knocked out, had no effect on the extent of injury caused by stroke.

**Conclusions—:**

These data suggest that the NLRP3 pathway does not contribute to the inflammation exacerbating ischemic brain damage, contradicting several recent reports to the contrary.

## Abstract 2

Supplemental Digital Content is available in the text.

Inflammation is a protective host response required for resistance to infection, though in response to tissue injury inflammation can also contribute to damage.^1^ Inflammatory cytokines associated with damaging inflammatory responses include members of the IL (interleukin)-1 family, IL-1α, and IL-1β.^1^ Evidence from preclinical models of stroke suggests IL-1 contributes to a worsening of ischemic brain injury.^2^ IL-1 activation and release from activated immune cells is regulated by a protein complex called the inflammasome.

Inflammasomes are cytosolic multimeric protein complexes formed in inflammatory cells in response to infection and injury.^3^ The most studied inflammasome is composed of the cytosolic PRR (pattern recognition receptor) NLRP3 (NACHT [NAIP (neuronal apoptosis inhibitor protein), C2TA (class 2 transcription activator, of the MHC), HET-E (heterokaryon_incompatibility) and TP1 (telomerase-associated protein 1)], LRR [leucine-rich repeat] and PYD [PYRIN] domains-containing protein 3). We previously reported ischemic brain injury developed independently of the NLRP3 inflammasome yet was modified in mice deficient in the PRRs NLRC4 (NOD-[nucleotide-binding oligomerization domain], LRR- and CARD [caspase activation and recruitment domain]-containing 4), AIM2 (absent in melanoma 2), or the inflammasome adaptor molecule ASC (apoptosis-associated speck-like protein containing a CARD).^4^ However, this is controversial and other groups have published correlations and associations with NLRP3 and a worsening of ischemic brain injury.^5–8^ Here, we sought to definitively determine the importance of NLRP3 to ischemic stroke using FeCl_3_ to induce thrombi formation in cerebral vessels and cause cerebral ischemia,^9^ with both genetic and pharmacological approaches to inhibit NLRP3.

## Methods

The data that support the findings of this study are available from the corresponding author on reasonable request.

### Animals

Experiments were performed on an in-house colony of 12- to 16-week-old male mice (N=72) on a C57BL/6 background (wild type [WT], NLRP3^−/−^^10^) at the University of Manchester. The NLRP3 deletion is whole organism–specific rather than cell-specific, which should be considered when interpreting data in such knockout studies. The experiments were performed on littermate controls except where stated and Figure I in the online-only Data Supplement which used mice maintained as homozygotes. Animals were allowed free access to food and water and maintained under temperature-, humidity-, and light-controlled conditions. All animal procedures adhered to the UK Animals (Scientific Procedures) Act (1986), and experiments were performed in accordance with ARRIVE (Animal Research: Reporting of In Vivo Experiments) guidelines, with researchers blinded to treatment or genotype.

### Model of Thrombotic Stroke

Thrombi formation and cerebral ischemia were performed using the FeCl_3_ method as described previously.^9^ Mice were anesthetized with 5% isoflurane, placed in a stereotaxic device, and maintained under anesthesia with 2.5% isoflurane in a 70%:30% mixture of N_2_O/O_2_. A small craniotomy (1 mm diameter) was performed on the parietal bone to expose the right middle cerebral artery (MCA). A Whatman filter paper strip soaked in FeCl_3_ (30%, Sigma) was placed on the dura mater on top of the MCA for 5 minutes.^9^ Cerebral blood flow in the MCA territory was measured continuously by laser Doppler flowmetry (Oxford Optronix).

### Pharmacological Treatment

MCC950 (Sigma) or saline were injected 30 minutes after onset of artery occlusion. Injections were intraperitoneal (100 µL, 50 mg/kg) or intracerebroventricular (1 µL, 10 µg) at the following stereotaxic coordinates: Bregma 0, lateral: −1 mm, dorso-ventral: −1.8 mm.

### Real-Time Quantitative Polymerase Chain Reaction

Total RNAs were extracted from samples with TRIzol Reagent (Thermo Fisher) according to the manufacturer. RNA (1 µg) was converted to cDNA using M-MLV (Moloney Murine Leukemia Virus) Reverse Transcriptase (Thermo Fisher). Quantitative polymerase chain reaction (PCR) was performed using Power SYBR Green PCR Master Mix (Thermo Fisher) in 384-well format using a 7900HT Fast Real-Time PCR System (Applied Biosystems). Three microliters of 1:50 diluted cDNA was loaded with 200 mmol/L of primers in triplicate. Data were normalized to the expression of the housekeeping gene *Hmbs*. Specific primers were designed using Primer3Plus software (http://www.bioinformatics.nl/cgi-bin/primer3plus/primer3plus.cgi). Primers used were: *Nlrp3* Forward—GCCCAAGGAGGAAGAAGAAG, *Nlrp3* Reverse—TCCGGTTGGTGCTTAGACTT, *Aim2* Forward—AAGAGAGCCAGGGAAACTCC, *Aim2* Reverse—CACCTCCATTGTCCCTGTTT, *Nlrc4* Forward—GTGACAGGCCTCCAGAACTT, *Nlrc4* Reverse—CCAAGCTGTCAATCAGACCA, *Pycard* Forward—TGCTTAGAGACATGGGCTTACA, *Pycard* Reverse—ACTCTGAGCAGGGACACTGG, *Casp1* Forward—CATTTGTAATGAAGACTGCTACCTG, *Casp1* Reverse—GATGTCCTCCTTTAGAATCTTCTGT, *Gsdmd* Forward—TGCAGATCACTGAGGTCCAC, *Gsdmd* Reverse—GCCTTCACCCTTCAAGCATA, *Il1b* Forward—AACCTGCTGGTGTGTGACGTTC, *Il1b* Reverse—CAGCACGAGGCTTTTTTGTTGT, *Il18* Forward—GACTCTTGCGTCAACTTCAAGG, *Il18* Reverse—CAGGCTGTCTTTTGTCAACGA, *Tnfa* Forward—GCCTCTTCTCATTCCTGCTTG, *Tnfa* Reverse—CTGATGAGAGGGAGGCCATT, *Il1a* Forward—TCTCAGATTCACAACTGTTCGTG, *Il1a* Reverse—AGAAAATGAGGTCGGTCTCACTA. Expression levels of genes of interest were computed as follows: relative mRNA expression=E^−(Ct of gene of interest)^/ E^−(Ct of housekeeping gene)^, where Ct is the threshold cycle value and E is efficiency.

### Flow Cytometry

Single-cell suspensions were prepared by digestion of brain tissue for 45 minutes at 37°C with 50 U/mL Collagenase (Gibco), 0.5 U/mL Dispase II (Gibco) and 200 U/ml DNase I (Roche) in Hank balanced salt solution, and red blood cells were lysed (Sigma). Myelin was removed by centrifugation in a 30% percoll solution (GE Healthcare). Cells were incubated with Fc block (anti-CD16/CD32 and rat serum) and surface-stained with fluorescence-conjugated anti-CD11b (M1/70), anti-Ly6G (1A8), anti-CD45.2 (104), and anti-CD64 (X54-5/7.1). Cells were then fixed (20 minutes, 4°C) with IC fixation buffer (eBioscience) before staining with APC (allophycocyanin)-eFluor780-conjugated antibody to mouse IL-1β (NJTEN3), or APC-eFluor780-conjugated Rat IgG1κ antibody (isotype- control), in permeabilization buffer (eBioscience) before acquisition. Live/Dead Aqua (Life Technologies) was used for exclusion of dead cells from analysis. Samples were analyzed by flow cytometry with an LSR II (Becton-Dickinson), and cells were characterized with FlowJo software. Cells were identified by expression of surface markers as follows: neutrophils–CD45^hi^/CD11b^hi^/Ly6G^hi^, monocytes–CD45^hi^/CD11b^hi^/Ly6C^hi^, and macrophages/microglia–CD45^int-hi^/CD11b^hi^/Ly6G^-^/Ly6C^-^/CD64^+^.

### Tissue Processing and Measurement of Infarct Volumes

Anesthetized mice were transcardially perfused with cold heparinized saline followed by 100 mL of fixative (PBS 0.1 mol/L, pH 7.4 containing 4% paraformaldehyde). Brains were removed, postfixed (24 hours), and cryoprotected (sucrose 20% in PBS; 24 hours) before freezing in optimal cutting temperature compound (PFM Medical UK, Ltd). Cryostat-cut sections (10 μm) were stained with Cresyl violet. Lesion volume was analysed using ImageJ and calculated as the sum of every lesion area multiplied by the distance between each section (0.4 mm).

### Immunohistochemistry

For immunostains, brain sections from littermate WT-, NLRP3^−/−^-, and MCC950-treated WT mice perfused fixed as above were dried for 24 hours before undergoing heat-mediated antigen retrieval in Tris-EDTA pH 8.6 solution for 20 min in a water bath set to 97.5°C. Sections were then incubated with rat–anti-Ly6G (1.25 μg/mL, 1A8, Biolegend) and goat–anti-IL-1β (1 μg/mL, AF-401-NA; RnDSystems) antibodies overnight at 4°C. Ly6G was labeled using donkey–anti-rat Alexa Fluor 647 (10 µg/mL; Abcam), IL-1β signal was amplified with Tyramide SuperBoostTM (Thermo Fisher) using biotinylated horse–anti-goat IgG (7.5 µg/mL, BA-9500; Vector) secondary antibody. Cellular IL-1β signal likely represents pro–IL-1β. Low magnification images were collected on an Olympus BX63 upright microscope, and high magnification images were taken on an Axio Imager.M2 Upright (Zeiss) microscope. Quantitative analysis was performed on 3 low magnification images of the lesion taken from 3 different coronal brain slices. Images were processed using ImageJ. The cells were then were counted using the analyse particles function, and colocalization analysis performed using the DiAna plugin.^11^

### Western Blotting of Isolated CD11b Positive Cells

Littermate WT and NLRP3^−/−^ mice were transcardially perfused with saline 24 hours after stroke. Brains were then removed and digested into a single-cell suspension and myelin removed (as described above), and cells were isolated by magnetic-activated cell sorting using CD11b+ magnetic beads (Miltenyi). Positive and negative CD11b+ cells were lysed in Tris-Triton buffer (150 mmol/L NaCl, 1% [vol/vol] Triton X-100, 50 mmol/L Tris, pH 7.5), supplemented with a protease inhibitor mixture. Cell lysates were separated by Tris-glycine SDS/PAGE and then transferred onto polyvinylidene fluoride (PVDF) membranes at 25 V using a semidry Trans-Blot Turbo system (Bio-Rad). Membranes were blocked in 2.5% (wt/vol) BSA in PBS, 1% (vol/vol) Tween 20 before being incubated with indicated primary antibodies at 4°C overnight. Membranes were then labeled with horseradish peroxidase–tagged secondary antibodies and visualized with Amersham ECL detection reagent (GE Healthcare). Western blot images were captured digitally using a G:Box Chemi XX6 (Syngene). Specific antibodies were used targeting mouse IL-1β (AF-401; R&D), ASC (D2W8U, Cell Signalling Technology), caspase-1 p10 (EPR16883; Abcam), and β-actin (Sigma).

### Statistical Analysis

Data are presented as mean values±SEM overlaid with individual data points. Sample size was calculated by power analysis using a significance level of α=0.05 with 80% power to detect statistical differences. Levels of significance were *P*<0.05, *P*<0.01, *P*<0.001. Statistical analyses were performed using GraphPad Prism (version 7). Quantitative PCR data were analysed using a 1-way ANOVA with Tukey post hoc comparisons. Flow cytometry and lesion volumes were analysed using 2-tailed *t* test.

## Results

**Figure 1.**
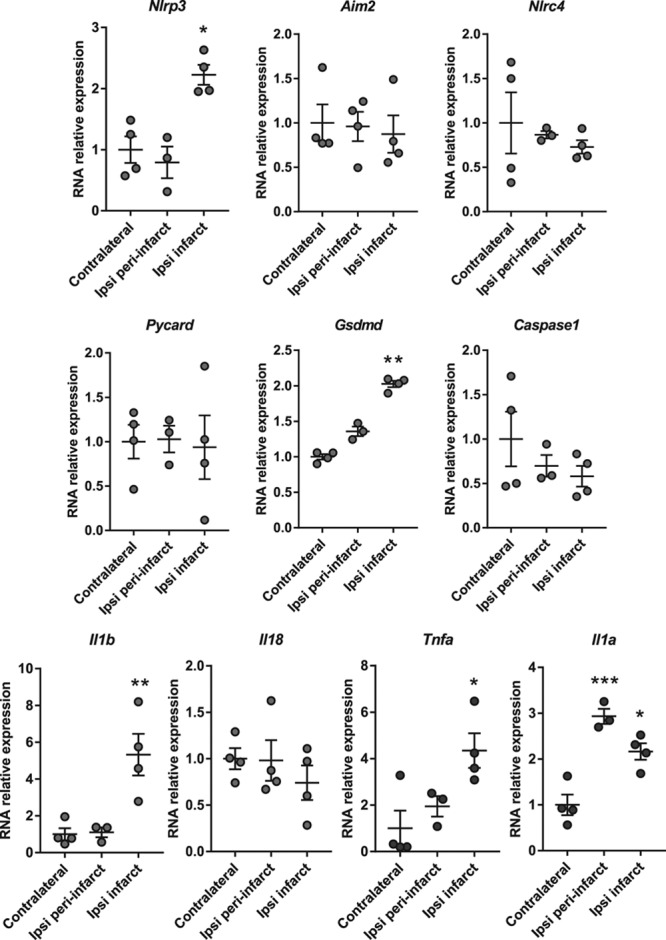
Inflammasome and cytokine expression 24 h after stroke. mRNA levels (normalized to contralateral tissue) of inflammasome components and cytokines in the contralateral, peri-infarct, and infarct areas at 24 h (n=3/4 mice, *P<0.05, **P<0.01, ***P<0.001, 1-way ANOVA followed by Tukey post hoc test).

Using the FeCl_3_ model of ischemia, mice were subjected to stroke, brains were removed 24 hours after stroke, and infarct, peri-infarct, and contralateral areas dissected and analysed for gene expression by real-time quantitative PCR (Figure 1). A significant increase in the expression of *Nlrp3*, *Gsdmd*, *Il1b*, *Il1a*, and *Tnfa* were measured in infarcted tissue (Figure 1). No differences were observed for *Aim2*, *Nlrc4*, *Pycard* (ASC), *Casp1*, or *Il18* expression. These data are consistent with previous reports showing increased expression of the NLRP3 system and other markers of inflammation in the injured tissue.^5,6,12,13^

**Figure 2.**
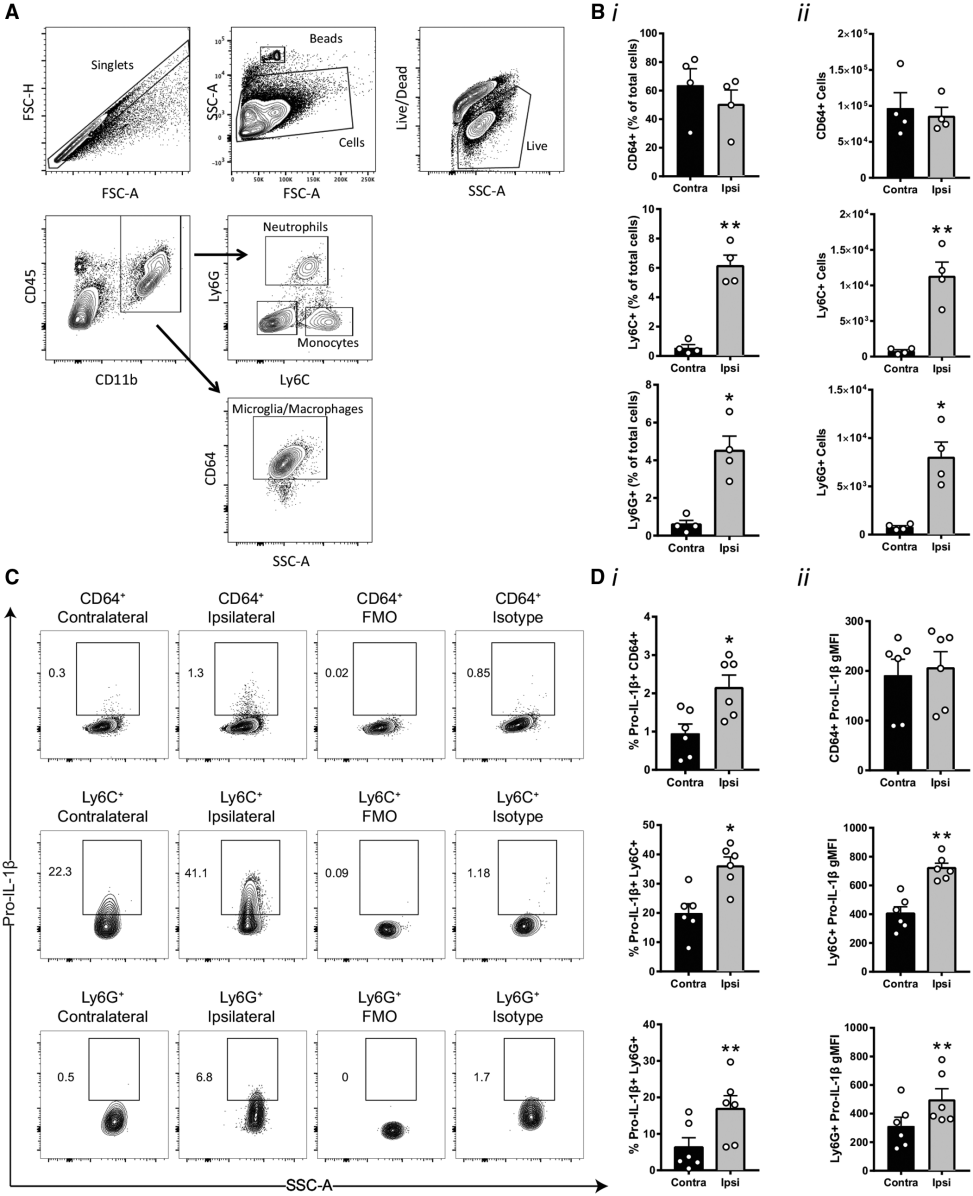
Immune cell recruitment and pro–IL (interleukin)-1β expression in immune cells 24 h after stroke. **A**, Gating strategy and flow cytometry analysis of microglia/macrophages CD45^int-hi^/CD11b^hi^/CD64^hi^, monocytes CD45^hi^/CD11b^hi^/Ly6C^hi^, and neutrophils CD45^hi^/CD11b^hi^/Ly6G^hi^ in the ipsilateral hemisphere. **B**, (i) Percentage of microglia/macrophages, monocytes, and neutrophils vs the total number of live cells in the contralateral and ipsilateral hemisphere. **B**, (ii) Absolute number of microglia/macrophages, monocytes, and neutrophils in the contralateral and ipsilateral hemisphere (n=4 mice, *P<0.05, **P<0.01, analysed by 2-tailed paired *t* test). **C**, Gating strategy analysis and flow cytometry analysis of pro–IL-1β 24 h after stroke. **D**, (i) Percentage of pro–IL-1β positive cells and (**D**, ii) geometric mean fluorescence intensity of pro–IL-1β staining in microglia/macrophages, monocytes, and neutrophils (n=6 mice, *P<0.05, **P<0.01, analysed by 2-tailed paired *t* test). FMO indicates fluorescence minus one; FSC-A, forward scatter–area; FSC-H, forward scatter–height; SSC-A, side scatter–area.

Activated microglia/macrophages and infiltrating immune cells (ie, monocytes and neutrophils) are present in the infarct and in the peri-infarct area after ischemic stroke.^14^ Previous research showed microglial IL-1β expression 24 hours after stroke.^13^ NLRP3 is also described to be expressed in infiltrating cells at a similar time point.^12^ To further characterize the inflammatory response occurring after ischemia, we used flow cytometry of brain homogenates to measure microglia and immune cell infiltrates. We identified defined cellular subsets based on specific epitope expression and quantified infiltrating neutrophils (CD45^hi^/CD11b^hi^/Ly6G^hi^), monocytes (CD45^hi^/CD11b^hi^/Ly6C^hi^), and macrophages/microglia (CD45^int-hi^/CD11b^hi^/CD64^hi^; Figure 2A). There were no increases in numbers of macrophages/microglia in the injured ipsilateral hemisphere when measured as a percentage of total immune cells (Figure 2Bi), or when analysed as total cell number Figure 2Bii). However, increases in monocytes and neutrophils were measured in the ipsilateral hemisphere after stroke (Figure 2Bi and 2Bii). IL-1β expressing cell populations were measured by flow cytometry (Figure 2C). Here, there was significantly increased IL-1β expression in macrophages/microglia, monocytes, and neutrophils (Figure 2D). Given that the expressed IL-1β was cellular, it was most likely pro–IL-1β. Together, these data show a marked inflammatory response in the brain after ischemia and significant upregulation of the NLRP3 system.

**Figure 3.**
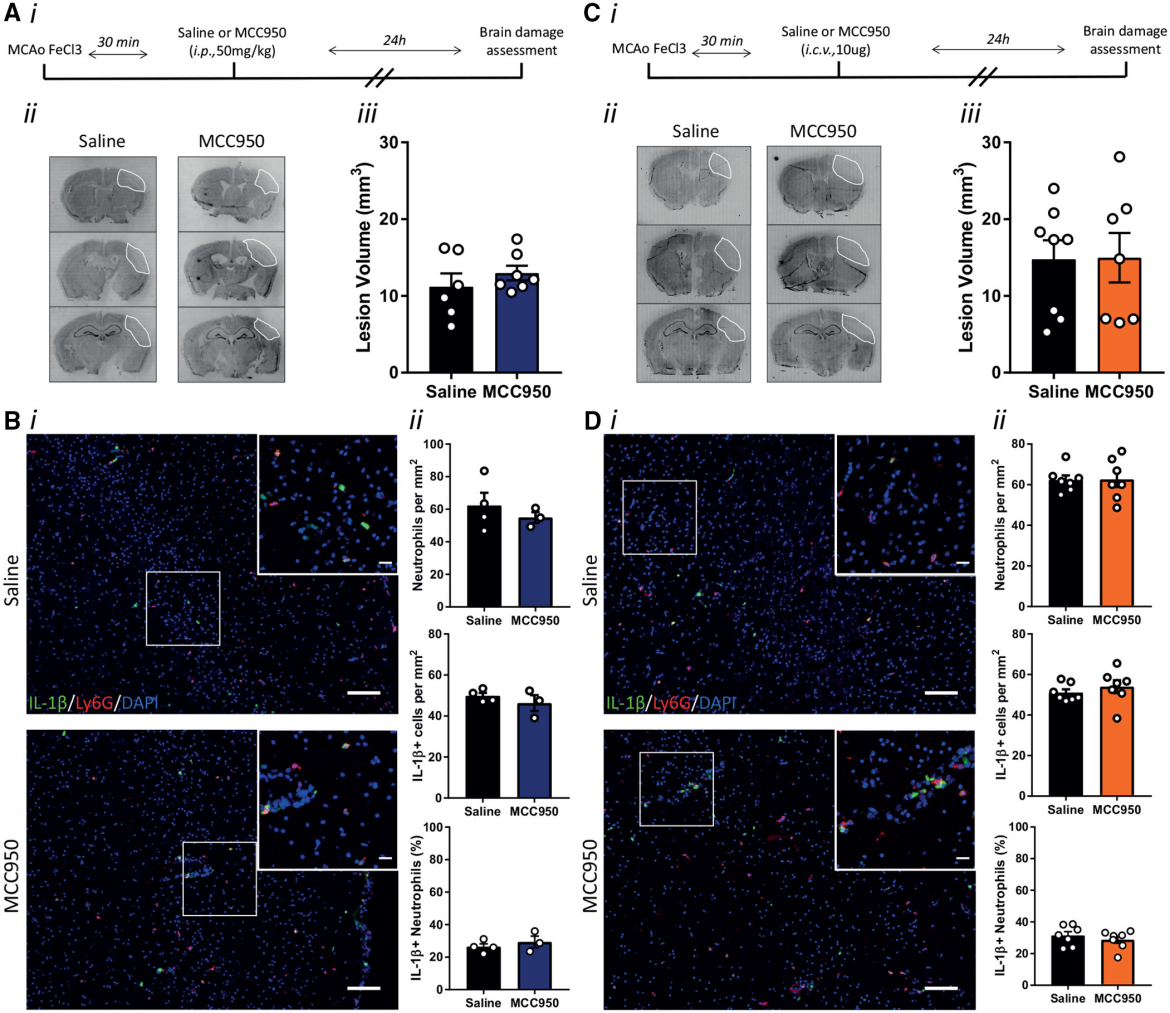
Influence of NLRP3 (NACHT, LRR and PYD domains-containing protein 3) inhibition on brain damage after stroke. **A**, (i) Schematic representation of experiment. **A**, (ii) representative cresyl violet staining and (iii) lesion volume quantification 24 h after stroke in mice treated with MCC950 (50 mg/kg) or saline by intraperitoneal injection 30 min after the stroke onset (n=6/group). **B**, (i) Representative immunostaining of IL (interleukin)-1β (green), neutrophils (Ly6G, red) and 4’,6-diamidine-2’-phenylindole dihydrochloride (DAPI; blue) in the infarct 24 h after stroke (scale bar in the large image is 100 μm and in the inset is 20 μm). **B**, (ii) Numbers of neutrophils and IL-1β positive cells per mm^2^, and % IL-1β positive neutrophils in the infarct 24 h after stroke plus and minus intraperitoneal MCC950 (n=3–4/group). **C**, (i) Schematic representation of the experiment. **C**, (ii) Representative cresyl violet staining and (iii) lesion volume quantification 24 h after stroke in mice treated with MCC950 (10 μg) or saline by intracerebroventricular injection 30 min after stroke onset (n=7–8/group). **D**, (i) Representative immunostaining of IL-1β (green), neutrophils (Ly6G, red) and DAPI (blue) in the infarct 24 h after stroke (Scale bar in the large image is 100 μm, and in the inset is 20 μm). **D**, (ii) Numbers of neutrophils and IL-1β positive cells per mm^2^, and % of IL-1β positive neutrophils in the infarct 24 h after stroke plus and minus intracerebroventricular MCC950 (n=7/group). Stroke was induced by middle cerebral artery occlusion (MCAo) using FeCl_3_.

**Figure 4.**
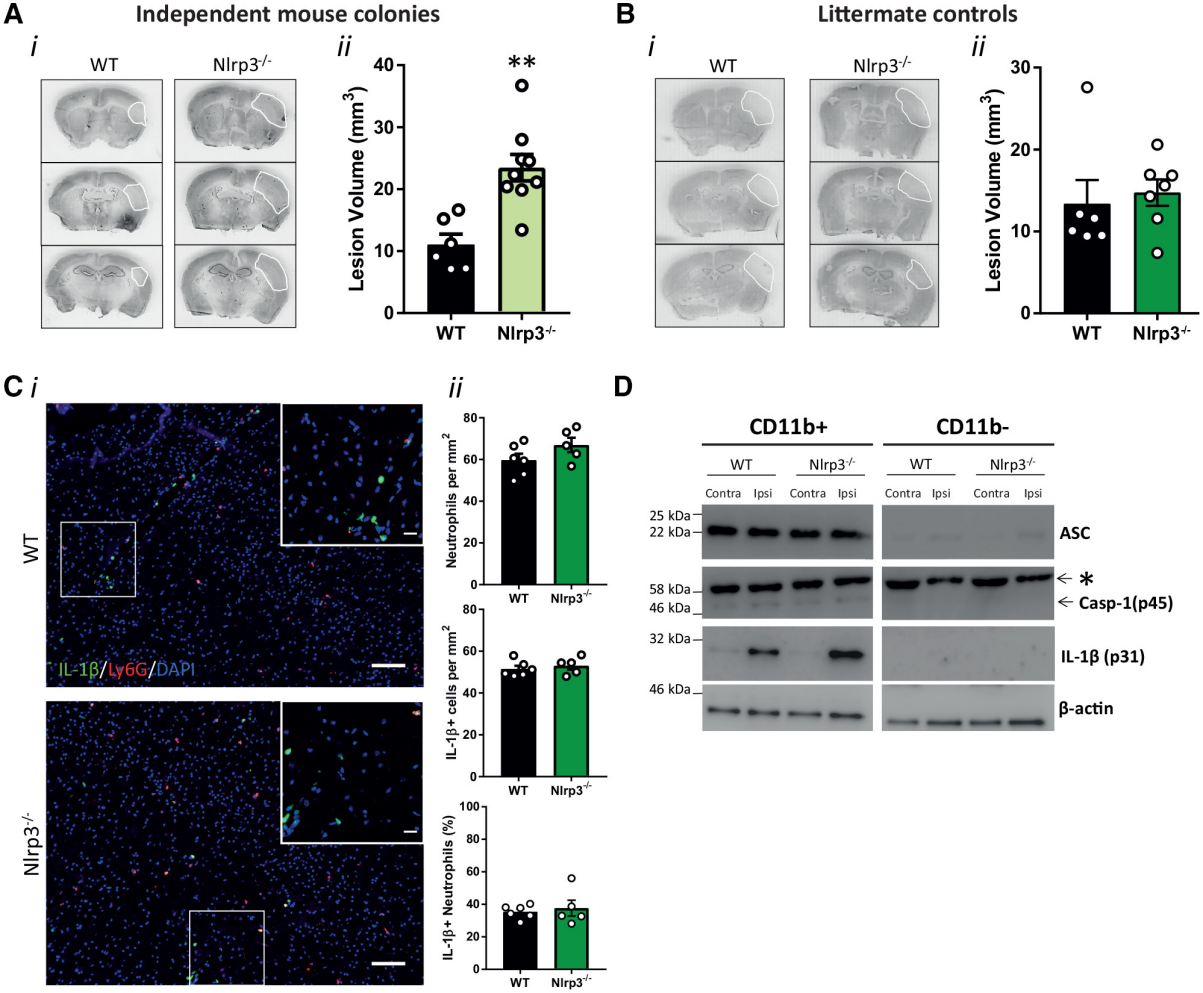
Influence of NACHT, LRR and PYD domains-containing protein 3 (NLRP3) gene deletion on brain damage after stroke. **A**, (i) Representative cresyl violet staining and (ii) lesion volume quantification 24 h after stroke in wild-type (WT) and nonlittermate NLRP3^−/−^ mice (n=6–9/group; **P<0.01, analysed by 2-tailed unpaired *t* test). **B**, (i) Representative cresyl violet staining and (ii) lesion volume quantification 24 h after stroke in WT and littermate NLRP3^−/−^ mice (n=6–7/group; ns denotes nonsignificant, analysed by 2-tailed unpaired *t* test). **C**, (i) Representative immunostaining of IL (interleukin)-1β (green), neutrophils (Ly6G, red), and 4’,6-diamidine-2’-phenylindole dihydrochloride (DAPI; blue) in the infarct 24 h after stroke (scale bar in the large image is 100 μm, and in the inset is 20 μm). **C**, (ii) Numbers of neutrophils and IL-1β positive cells per mm^2^ and % IL-1β positive neutrophils in the infarct 24 h after stroke in WT and littermate NLRP3^−/−^ mice (n=5–6/group). **D**, ASC (an apoptosis-associated speck-like protein containing a CARD), Casp (caspase)-1, IL-1β, and β-actin Western blot of cell lysates from magnetic bead cell isolation of myeloid cells (CD11b+) and nonmyeloid cells (CD11b-) from the ipsi and contralateral hemisphere 24 h after stroke (n=3). *Nonspecific band.

We recently evaluated a panel of the best literature compounds for their effects on inhibition of ASC speck formation and IL-1β release, hallmarks of NLRP3 inflammasome activation.^15^ The sulfonylurea containing MCC950 was the most potent and selective inhibitor of NLRP3 and retained its inhibitory activity in primary adult microglia.^15^ MCC950 delivered intraperitoneally did not affect lesion volume after thrombotic stroke (Figure 3A). MCC950 also failed to inhibit the recruitment of neutrophils, IL-1β expression, or neutrophil IL-1β expression as measured by immunohistochemistry (Figure 3B). To ensure that MCC950 reached the required site of action an additional experiment with central (lateral ventricle) injection of MCC950 was performed. Again, ischemic brain injury was unaffected by MCC950 treatment (Figure 3C). As with peripheral MCC950 administration, central administration of MCC950 had no effect on the recruitment of neutrophils, IL-1β expression, or neutrophil IL-1β expression as measured by immunohistochemistry (Figure 3D). To validate these pharmacological data, thrombotic stroke was first induced in NLRP3^−/−^ mice that were maintained as a homozygote colony, and age- and sex-matched C57BL/6 mice. Unexpectedly, infarct volume was significantly enhanced in the NLRP3^−/−^ mice (maintained as homozygotes) compared with WT (Figure 4A). RNA-sequencing analysis of isolated adult microglia from these WT and NLRP3^−/−^ mice showed that, at baseline, there were over 700 differentially expressed genes (Figure I in the online-only Data Supplement) suggesting that maintenance as a homozygote colony of the mice resulted in genetic drift. We, therefore, repeated the experiment using littermate WT and NLRP3^−/−^ mice bred from heterozygote breeding pairs. In this experiment, there was no difference in ischemic brain injury between WT and NLRP3^−/−^ mice (Figure 4B). These data are consistent with our previous work in the filament middle cerebral artery occlusion model,^4^ and provide robust evidence for a lack of involvement of the NLRP3 inflammasome in ischemic brain injury, contrary to other recently published data,^5–8^ and highlight the pitfalls of using homozygote knockout mouse colonies. Analysis of neutrophil recruitment and IL-1β expression in the brains of WT versus littermate NLRP3^−/−^ mice revealed no difference in the recruitment of neutrophils, IL-1β expression, or neutrophil IL-1β expression consistent with the MCC950 experiments presented in Figure 3 (Figure 4C). Magnetic bead separation of myeloid and nonmyeloid cell populations from the ipsi and contralateral hemispheres of WT and littermate NLRP3^−/−^ mice followed by Western blotting revealed that only the myeloid cell population expressed detectable inflammasome adaptor protein ASC and caspase-1, and only myeloid cells isolated from the injured ipsilateral hemisphere expressed pro–IL-1β, and this was unchanged between WT and NLRP3^−/−^ mice (Figure 4D).

## Discussion

Here, we report that ischemic brain injury was not reduced by specific inhibition of NLRP3 with MCC950 or in NLRP3^−/−^ mice. These data provide evidence that NLRP3, the canonical sensor of sterile injury, is not involved in the acute phase of ischemic stroke.

NLRP3 has been described to be involved in sterile brain injury and diseases, such as Alzheimer disease, multiple sclerosis, and traumatic brain injury.^16^ However, in the context of stroke, there is some controversy over its involvement in increasing infarct volume. Previous studies have shown that mice deficient in NLRP3 have a reduction in brain damage^6^ or that NLRP3 inhibition with an antibody^5^ or with MCC950^8^ is protective. In contrast, we previously reported mice deficient in NLRP3 had no reduction in brain injury caused by the filament model of middle cerebral artery occlusion.^4^ The involvement of inflammation in ischemic injury, and in particular IL-1–dependent inflammation is less equivocal with studies reporting that genetic deletion of IL-1, or blocking the IL-1 receptor with the naturally occurring IL-1Ra (IL-1 receptor antagonist) is protective.^17,18^ In this study, we chose to use a more clinically relevant model of stroke using FeCl_3_ to induce thrombosis of the MCA.^9^ Although there was a marked increase in inflammation using the FeCl_3_ model, there was no reduction in ischemic damage using the specific and potent NLRP3 inhibitor MCC950, or in the littermate NLRP3^−/−^ experiment, confirming our previous report that NLRP3 does not contribute to ischemic brain injury.^4^ Expression of IL-1β and myeloid cell recruitment was also independent of NLRP3. However, we do not exclude the likelihood that NLRP3 contributes to other cardiovascular events. The positive result of the CANTOS trial (Canakinumab Anti-Inflammatory Thrombosis Outcomes Study) highlights the importance of IL-1β in the risk of recurrent cardiovascular events.^19^ Moreover, NLRP3 has been demonstrated to contribute to the vascular inflammatory response driving atherosclerosis,^20^ suggesting that NLRP3 could contribute to an increased risk of ischemic stroke.

These data also highlight a cautionary note for using homozygote knockout mouse colonies. We observed significant genetic changes in pure microglia isolated from age- and sex-matched, cohoused, adult WT and NLRP3^−/−^ mice which may explain the observed increase in infarct volume. In studies using nonlittermate NLRP6 and ASC knockout animals, NLRP6 and ASC were reported to be regulators of the gut microbiome and influence the severity of colitis.^21^ However, subsequent studies using littermate controls find that NLRP6 and ASC do not regulate the composition of gut microbiota and that maternal inheritance and long-term separate housing are the biggest nongenetic confounders when studying the microbiome.^22,23^ These studies^22,23^ and our own argue the necessity of littermate-controlled experiments when studying mechanisms of innate immunity.

## Summary

In summary, this study suggests that although there is a marked inflammatory response after ischemic stroke, NLRP3-dependent inflammation is not involved in exacerbation of the injury and thus does not represent a therapeutic target for ischemic brain injury.

## Acknowledgments

We are grateful to Genentech for the NLRP3 (NACHT, LRR and PYD domains-containing protein 3)^−/−^ mice. We are also grateful to Jack Rivers-Auty (University of Manchester) who advised on the statistical analysis, core-facility service at the University of Manchester, Biological Services Facility, Genomic Technologies Facility, Bioimaging Facility, and Histology Facility for expert guidance and use of facilities.

## Sources of Funding

This work was funded by the Medical Research Council (grant number MR/N003586/1 to Drs Brough, Allan, and Lemarchand; MC_PC_16033 to Drs Brough and Haley) and Wellcome Trust to Dr Allen (106898/A/15/Z).

## Disclosures

None.

## Supplementary Material
