# Supplementary material for: Extent of Ischemic Brain Injury After Thrombotic Stroke Is Independent of the NLRP3 (NACHT, LRR and PYD Domains-Containing Protein 3) Inflammasome
Source: Stroke. 2019 Apr 8;50(5):1232–9. doi: 10.1161/STROKEAHA.118.023620 (PMC6485300; doi:10.1161/STROKEAHA.118.023620)
Supplement: Supplementary file 1 [file str-50-1232-s001.pdf]

SUPPLEMENTAL MATERIAL

Extent of ischemic brain injury following thrombotic stroke is independent of the NLRP3 inflammasome

Eloise Lemarchand, PhD[1,3], Jack Barrington BSc[1,3], Alistair Chenery, PhD[2,3], Michael Haley, PhD[2,3], Graham Coutts, PhD[1,3], Judith E. Allen, PhD[2,3], Stuart M. Allan, PhD[1,3,*, #], David Brough, PhD[1,3,*,#]

Supplemental Methods

Supplemental Figure I

Supplemental Methods

RNA-seq analysis of isolated microglia

Mice (NLRP3$^{-/-}$ bred as homozygotes, and wild type, with both genotypes co-housed) were transcardially perfused with ice-cold Hank's balanced-salt solution. Brains were then digested into a single-cell suspension and myelin removed (as described above), and microglia isolated by magnetic-activated cell sorting using Cd11b+ magnetics beads (Miltenyi). RNA was isolated using a Qiagen RNeasy Micro Kit. After confirming RNA integrity, RNA was sequenced on an Illumina HiSeq2500. The sequenced data was then trimmed using FastQC, and mapped into the murine genome. Raw count data was normalised in DESeq2, and corrected counts of genes compared pairwise between genotypes, with a correction for false discovery rate.

Supplemental Figure I

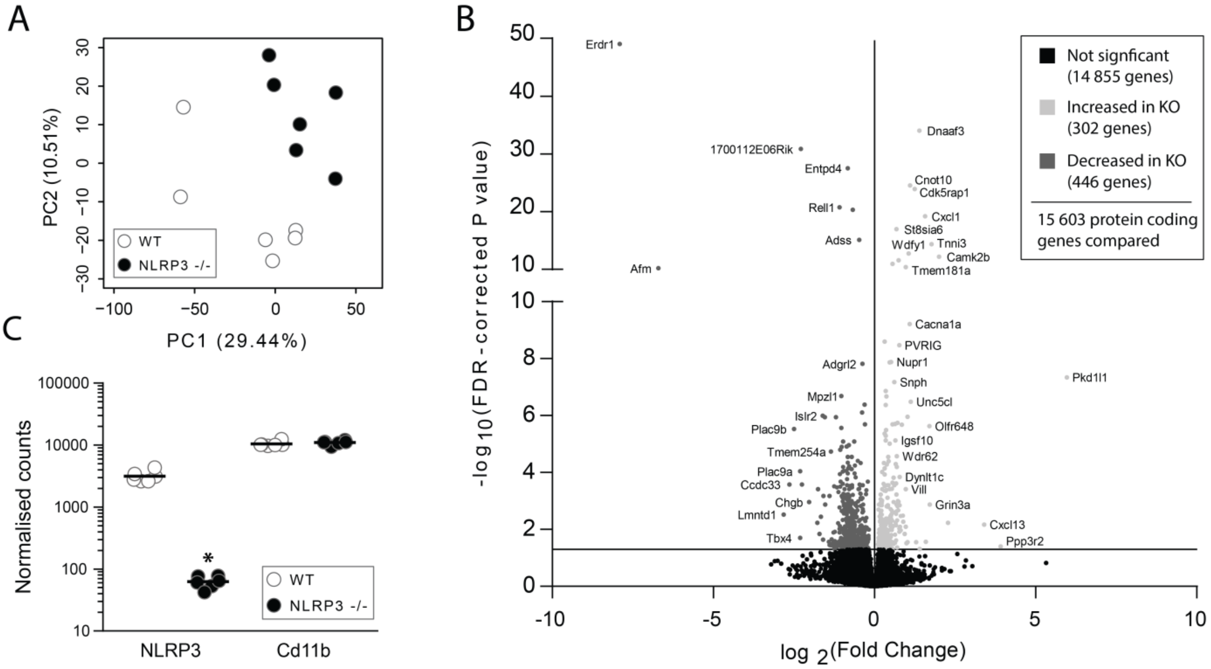

Supplemental Figure I: (A) Clear clustering of genotypes can be seen by principal components analysis. (B) Of the 22561 genes identified, 15603 were protein coding, with 708 of these differentially expressed between genotype. (C) NLRP3 knock out was confirmed, and cd11b (used to isolate microglia) was not differentially expressed (n=6 mice/genotype, differentially expressed genes identified by pair-wise comparison with correction for false-discovery rate).

*** Preclinical Checklist**

Preclinical Checklist: Prevention of bias is important for experimental cardiovascular research. ***This short checklist must be completed, and the answers should be clearly presented in the manuscript.*** *The checklist will be used by reviewers and editors and it will be published. See "Reporting Standard for Preclinical Studies of Stroke Therapy" and "Good Laboratory Practice: Preventing Introduction of Bias at the Bench" for more information.*

This study invovles animal models:
Yes

Experimental groups and study timeline

The experimental group(s) have been clearly defined in the article, including number of animals in each experimental arm of the study:	Yes
An account of the control group is provided, and number of animals in the control group has been reported. If no controls were used, the rationale has been stated:	Yes
An overall study timeline is provided:	No

Inclusion and exclusion criteria

A priori inclusion and exclusion criteria for tested animals were defined and have been reported in the article:	Yes

Randomization

Animals were randomly assigned to the experimental groups. If the work being submitted does not contain multiple experimental groups, or if random assignment was not used, adequate explanations have been provided:	Yes
Type and methods of randomization have been described:	No
Methods used for allocation concealment have been reported:	No

Blinding

Blinding procedures have been described with regard to masking of group/treatment assignment from the experimenter. The rationale for nonblinding of the experimenter has been provided, if such was not feasible:	Yes
Blinding procedures have been described with regard to masking of group assignment during outcome assessment:	Yes

Sample size and power calculations

Formal sample size and power calculations were conducted based on a priori determined outcome(s) and treatment effect, and the data have been reported. A formal size assessment was not conducted and a rationale has been provided:	Yes

Data reporting and statistical methods

Number of animals in each group: randomized, tested, lost to follow-up, or died have been reported. If the experimentation involves repeated measurements, the number of animals assessed at each time point is provided, for all experimental groups:	Yes
Baseline data on assessed outcome(s) for all experimental groups have been reported:	N/A
Details on important adverse events and death of animals during the course of experimentation have been provided, for all experimental arms:	N/A
Statistical methods used have been reported:	Yes
Numeric data on outcomes have been provided in text, or in a tabular format with the main article or as supplementary tables, in addition to the figures:	No

Experimental details, ethics, and funding statements

Details on experimentation including stroke model, formulation and dosage of therapeutic agent, site and route of administration, use of anesthesia and analgesia, temperature control during experimentation, and postprocedural monitoring have been described:	Yes
Different sex animals have been used. If not, the reason/justification is provided:	No

Statements on approval by ethics boards and ethical conduct of studies have been provided:	Yes
Statements on funding and conflicts of interests have been provided:	Yes

---

Date completed: 02/18/2019 09:32:20
User pid: 54959
