# Supplementary material for: Extent of Ischemic Brain Injury After Thrombotic Stroke Is Independent of the NLRP3 (NACHT, LRR and PYD Domains-Containing Protein 3) Inflammasome
Source: Stroke. 2019 Apr 8;50(5):1232–9. doi: 10.1161/STROKEAHA.118.023620 (PMC6485300; doi:10.1161/STROKEAHA.118.023620)
Supplement: Supplementary file 2 [file str-50-1232-s002.pdf]

American Heart Association | American Stroke Association®

Acknowledgment Permission Form

Journal Stroke
Manuscript Number STROKE/2018/023620
First Author Eloise Lemarchand
Title of Work Extent of ischemic brain injury following thrombotic stroke is independent of the NLRP3 inflammasome

Authors must provide written permission/approval from all individuals mentioned by name in the Acknowledgments section of a submitted manuscript. By signing this form, any and all acknowledged persons therefore state that they have read and approved the mention of their names in the Acknowledgment section of the aforementioned paper.

	Name	Signature	Date
Name (1)	Jack Rivers-Auty	Jack Rivers-Auty Digitally signed by Jack Rivers-Auty Date: 2018.12.18 08:18:55 Z	17/12/2018
Name (2)			
Name (3)			
Name (4)			
Name (5)			
Name (6)			
Name (7)			
Name (8)			
Name (9)			
Name (10)			
Name (11)			
Name (12)			
Name (13)			
Name (14)			
Name (15)			
Name (16)			
Name (17)			
Name (18)			
Name (19)			
Name (20)			
