# Supplementary figures and images for: Extent of Ischemic Brain Injury After Thrombotic Stroke Is Independent of the NLRP3 (NACHT, LRR and PYD Domains-Containing Protein 3) Inflammasome
Source: Stroke. 2019 Apr 8;50(5):1232–9. doi: 10.1161/STROKEAHA.118.023620 (PMC6485300; doi:10.1161/STROKEAHA.118.023620)

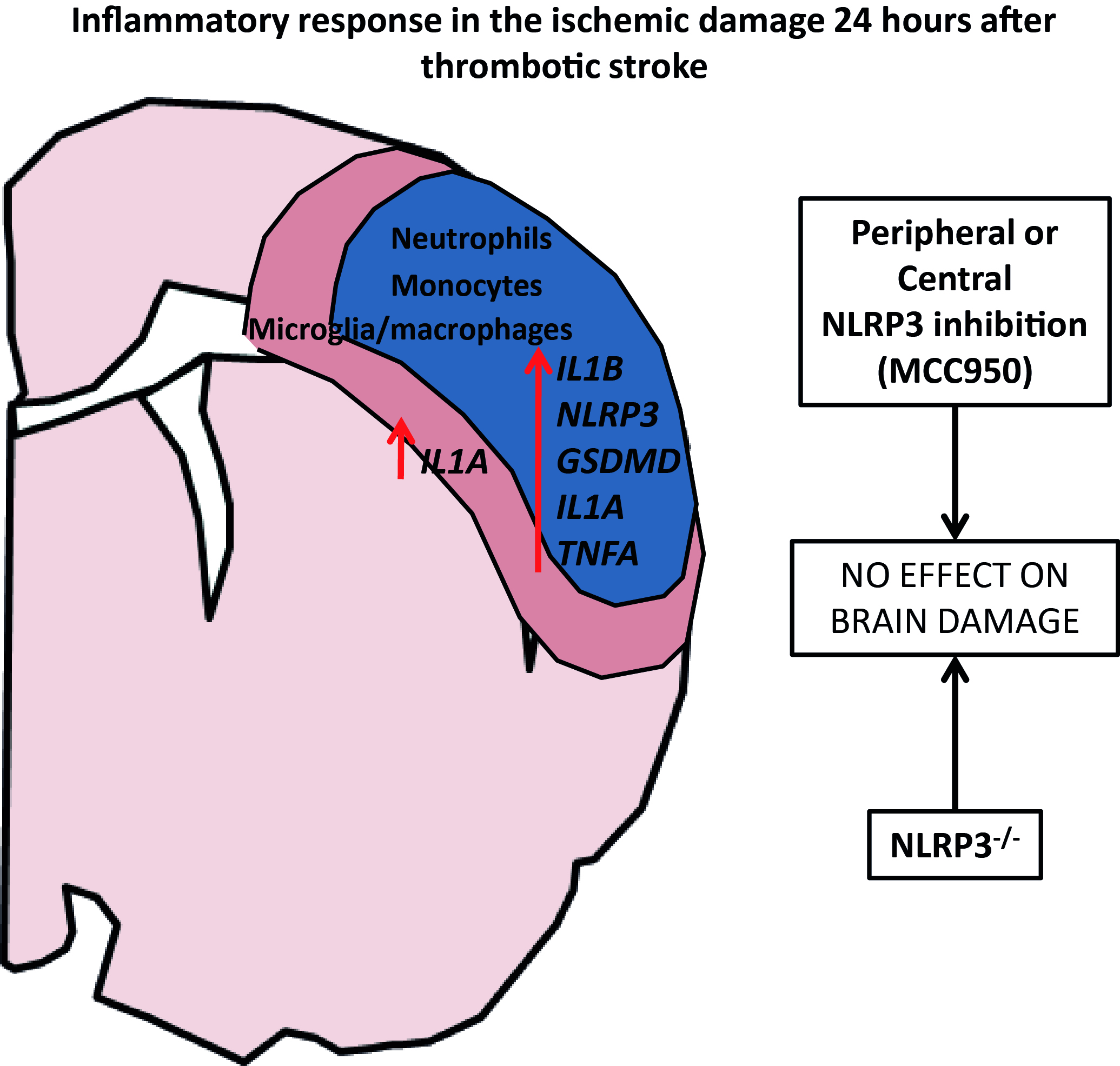

Supplement: Supplementary file 3 [file str-50-1232-s003.jpg]
